# ATR-FTIR Spectroscopy with Chemometrics for Analysis of Saliva Samples Obtained in a Lung-Cancer-Screening Programme: Application of Swabs as a Paradigm for High Throughput in a Clinical Setting

**DOI:** 10.3390/jpm13071039

**Published:** 2023-06-25

**Authors:** Francis L. Martin, Andrew W. Dickinson, Tarek Saba, Thomas Bongers, Maneesh N. Singh, Danielle Bury

**Affiliations:** 1 Biocel UK Ltd., Hull HU10 6TS, UK; mnsingh@biocel.uk; 2 Department of Cellular Pathology, Blackpool Teaching Hospitals NHS Foundation Trust, Whinney Heys Road, Blackpool FY3 8NR, UK; aw.dickinson@hotmail.com (A.W.D.); dr.saba@nhs.net (T.S.); thomas.bongers@nhs.net (T.B.); 3 Chesterfield Royal Hospital, Chesterfield Road, Calow, Chesterfield S44 5BL, UK

**Keywords:** ATR-FTIR spectroscopy, chemometrics, “dip” test, lung cancer, saliva, swab

## Abstract

There is an increasing need for inexpensive and rapid screening tests in point-of-care clinical oncology settings. Herein, we develop a swab “dip” test in saliva obtained from consenting patients participating in a lung-cancer-screening programme being undertaken in North West England. In a pilot study, a total of 211 saliva samples (*n* = 170 benign, 41 designated cancer-positive) were randomly taken during the course of this prospective lung-cancer-screening programme. The samples (sterile Copan blue rayon swabs dipped in saliva) were analysed using attenuated total reflection Fourier-transform infrared (ATR-FTIR) spectroscopy. An exploratory analysis using principal component analysis (PCA,) with or without linear discriminant analysis (LDA), was then undertaken. Three pairwise comparisons were undertaken including: (1) benign vs. cancer following swab analysis; (2) benign vs. cancer following swab analysis with the subtraction of dry swab spectra; and (3) benign vs. cancer following swab analysis with the subtraction of wet swab spectra. Consistent and remarkably similar patterns of clustering for the benign control vs. cancer categories, irrespective of whether the swab plus saliva sample was analysed or whether there was a subtraction of wet or dry swab spectra, was observed. In each case, MANOVA demonstrated that this segregation of categories is highly significant. A k-NN (using three nearest neighbours) machine-learning algorithm also showed that the specificity (90%) and sensitivity (75%) are consistent for each pairwise comparison. In detailed analyses, the swab as a substrate did not alter the level of spectral discrimination between benign control vs. cancer saliva samples. These results demonstrate a novel swab “dip” test using saliva as a biofluid that is highly applicable to be rolled out into a larger lung-cancer-screening programme.

## 1. Introduction

In a growing worldwide population, the (self-)management of chronic diseases, such as cancer, diabetes and neurodegenerative conditions, will become increasingly important. Critical to this will be a need for rapid and simple screening and/or diagnostic methodologies towards patient triage. Especially since the COVID-19 pandemic, hospital settings are over-burdened and there is an urgent need to develop approaches to allow routine testing to provide a rapid and informed indication, either in a home setting, in a primary care setting, such as a GP’s surgery, or at the entrance to A&E [1]. Such a methodology needs to be easy to implement, accurate in its output, readily interpretable for a non-expert, inexpensive, given the volume of testing required, repeatable and quick [2].

Vibrational spectroscopy, including attenuated total reflection Fourier-transform infrared (ATR-FTIR) spectroscopy, combined with chemometrics, has the potential to be translated to a variety of clinical settings [3]. The basis of this sensor-based approach is that a fingerprint spectrum can be derived from a biological sample based on its chemical bond composition; a reference range of what constitutes a benign control condition can be constructed and predictive analyses can suggest the likely outcome of spectra that fall outside this [4]. Readily accessible biofluids, such as blood plasma/serum, saliva or urine, are considered ideal for clinical implementation due to routine methods of collection, as well as minimal sample preparation. Biofluid-based ATR-FTIR spectroscopy approaches have been used for diagnosing, screening or monitoring the progression/regression in a variety of disease conditions [5,6]. We have previously shown the diagnostic capability of this approach to detect brain tumours, both primary and metastatic, from blood plasma with an accuracy of 88–100% [7]. It is also possible to employ saliva analysed using ATR-FTIR spectroscopy to distinguish from normal through to Barrett’s oesophagus, dysplasia up to adenocarcinoma. Within the normal vs. adenocarcinoma groups, this is with sensitivities from 89 to 100% and specificity of 60 to 100% [4].

Typically, the sample to be analysed needs to be placed on a substrate before it is applied to the sensor to facilitate infrared (IR) spectral acquisition, and there are several substrate types to facilitate this [8]. Alternatively, the sample, such as a biofluid (just 1 μL required), can be applied directly to the diamond sensor of an ATR-FTIR device [9]. In other cases, the sample can be applied to a substrate such as an aluminium-coated glass slide [10]. A biofluid in this case would need to be aliquoted onto a substrate in a discrete spot and then allowed to dry for a couple of hours. For the non-expert practitioner, this increases the handling of biological material and might be a potentially error-prone methodology. An approach pioneered by our group is the notion of a “dip” test whereby a sterile swab is placed in the biofluid in question and after mixing, is then applied to the sensor of the ATR-FTIR spectrometer [1]. Advantages of this approach are familiarity (amongst clinical staff such as nurses or doctors) due to similarities with other testing regimens, achievable consistency for the non-expert practitioner and ready ease-of-use in a typical clinical setting. A potential disadvantage is that the swab material will have its own underlying IR spectral signature, but we would contend that many substrates would give rise to an absorbance spectral profile in the biological fingerprint region. If this underlying “contaminating” spectral signature is consistent, it should remain possible to extract and interpret the overlying biological spectra and this should be sufficient for an interpretable screening and/or diagnostic test.

This investigation was nested in a prospective study of patients attending the Blackpool Targeted Lung Health Check, similar to others being carried out in the North of England [11]. These patients have been pre-selected based on multiple factors, including age and smoking history, to be deemed ‘at risk’ of lung cancer. Once they have undergone health checks, patients that trigger a low-radiation-dose computed tomography (CT) scan for further investigation will be consented to take part in this study. This was performed by the nurse undertaking the initial assessment and consenting them for involvement within the screening pilot. Once consented, patients were asked to provide saliva for testing by spitting into a sterile universal container. The saliva was tested on a portable IR spectrometer. Chemometric analysis, to develop predictive models to allow the determination of sensitivities and specificities for saliva for the diagnosis of lung cancer, was undertaken. This study was nested in a true clinical setting. It is not an artificially constructed scenario where one compares lung cancers vs. benign. This is a real-world setting wherein all the people coming into clinic are “at-risk”. This addresses the challenge clinicians face worldwide: how does one pick out the small number of disease states that require intervention from the large mass of individuals with complex co-morbidities?

Given the large-scale prevalence of lung cancer within the North West of England, and its selection to be part of the National Lung Cancer Screening pilot due to the high cancer inequality seen, this study was developed to run alongside this pilot. This allows us to test the potential benefit of this alternative technique, either as an aid to or in the replacement of the more intensive and expensive screening pilot. This study aims to determine if saliva can provide a useful screening tool for the detection of lung cancer, within a real-life clinical setting, to translate it into a clinically useful and viable diagnostic test, which benefits patients. Our initial objective, using a substantial subset of the study participants recruited into the trial, was to determine whether employing a swab as part of the methodology would give a robust and consistent approach for routine spectral analysis.

## 2. Materials and Methods

### 2.1. Lung-Cancer-Screening Programme and Participant Recruitment

This study was carried out in agreement with the Helsinki declaration and full ethical approval was obtained (HRA IRAS ref: 276081; REC ref: 20/PR/0390; London Bridge REC). All procedures and possible risks were explained to participants before they provided written consent. The study was nested in a prospective study of people invited to attend the National Lung Cancer screening pilot in the Blackpool area of North West England. These potential participants were pre-selected to be deemed ‘at risk’ of lung cancer, based on multiple factors including age and smoking history. Once they had undergone health checks, those participants that trigger a CT scan for further investigation were consented, if willing, to take part in this study. This was performed by the nurse undertaking the initial assessment and consent for involvement within the screening pilot. The rationale for this approach was to provide a mixture of both suspected cancer and non-cancer patients. All participants had a CT scan and those that exhibited no lung lesions were immediately assigned to the benign group. A visible lesion triggered an urgent oncology referral. Participants who underwent surgery were proven to have cancer following histopathology undertaken by a Consultant Histopathologist. A small number of participants had radiotherapy; these were also assigned as cancer. Additionally, some participants sent for oncology referral had benign lesions; these individuals were assigned to the benign group. All participants were followed for up to 2 years in order to validate these outcomes. A total of 211 saliva samples (*n* = 170 benign, 41 designated cancer positive) were randomly taken in the order in which they entered the clinic (i.e., there was no selection of participants in order to avoid bias) during the course of this prospective lung-cancer-screening programme.

### 2.2. Saliva Collection and Swab Analysis

For all participants, demographic data (age, gender, pre-existing medical conditions, symptoms, date of symptoms’ onset) were collected for NHS records; these will be accessible as the study progresses and more outcomes are known. Once consent has been given, participants were requested to provide saliva for testing by spitting into a sterile universal container. Samples were transported to the laboratory within 24 h where they were frozen at −20 °C until preparation for analysis. For the purpose of spectral analysis, a plain sterile rayon-tipped swab (Ref no.: 155C; Copan, Italy) was placed in the thawed (at room temperature) saliva sample to be tested and mixed, prior to spectral interrogation of the swab. The swab was applied directly to the ATR ZnSe crystal for spectral analysis—this was found to be an extremely convenient means of handling this biological material. Whilst there are contributing peaks from the swab, our objective was solely to develop a technique capable of giving a yes/no answer to the possibility, or not, of lung cancer being present.

### 2.3. ATR-FTIR Spectral Analyses of Swabs

**Figure 1:**
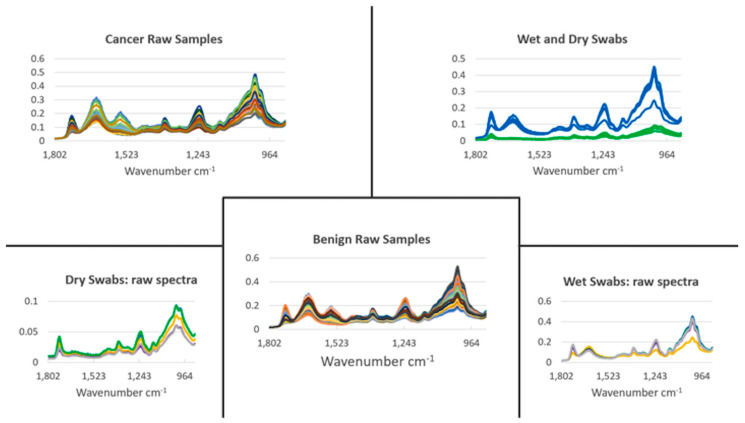
Raw mid-infrared spectra derived using ATR-FTIR spectroscopy. Saliva samples were obtained from consented participants in a lung-cancer-screening programme. Following transport to the laboratory, a sterile Copan blue rayon swab was dipped in the saliva sample, whereupon the swab was then analysed on the IR spectrometer. From each saliva sample, three independent spectral measurements were taken. For comparison, spectral measurements in triplicate from five separate swabs, wet (in milli-Q water; blue) or dry (green), are shown. In all measurements taken, only one obvious outlier was noted (in the benign control category) and this was excluded from subsequent analyses. *y*-axes are Absorbance (a.u.).

FTIR spectra data (wavenumber range 4000–650 cm^−1^) for each swab were obtained by directly placing the saliva swab on a portable Agilent Cary 630 FTIR Spectrometer equipped with an ATR ZnSe crystal (Agilent, Santa Clara, CA, USA) and Microlab PC software run from a dedicated computer laptop. Each whole spectrum contains 1798 points (1.86 cm^−1^ spectral resolution). For every ATR-FTIR spectroscopic measurement, three spectra were obtained from each saliva swab. Each swab analysis was performed with 32 co-additions, interspersed with 32 background scans. After each analysis, the swab was removed from the crystal and the crystal was cleaned with miliQ water and 70% alcohol, thus avoiding inter-sample contamination. Only a single swab analysis in the spectral dataset generated outliers (Figure 1).

### 2.4. Computational Analysis: Pre-Processing and Chemometrics

All data analytics were performed using python and available libraries. Spectral pre-processing for data analysis consisted of Savitzky–Golay (SG) smoothing (window of 7 points, 1st-order polynomial fitting) and 2nd derivative followed by vector normalization. SG smoothing corrects for random noise, 2nd derivative corrects for baseline distortions and vector normalization corrects for physical differences between samples such as thickness, light scattering and concentrations.

Principal component analysis (PCA) was used for exploratory analysis. PCA reduces the pre-processed spectral dataset into a small number of principal components (PCs), responsible for the majority of data variance. Each PC is composed of scores and loadings; the former is used to access similarity/dissimilarity patterns among samples and the latter to identify spectral features (wavenumbers), associated with class separation and, therefore, possible spectral biomarkers.

PCA models were built using the PCA software tool for python available on Github [12]. Further visualisation of the key discriminating wavenumbers is demonstrated in the form of a biplot produced in python using mpl toolkits (mpl_toolkits.mplot3d.axes3d.Axes3D at 0×1e215e3e580).

PCA is an unsupervised technique that reduces the spectral data space to PCs responsible for the majority of variance in the original dataset. Each PC is orthogonal to each other, where the first PC accounts for the maximum explained variance followed by the second PC and so on. The PCs are composed of scores and loadings, where the first represents the variance on sample direction, thus being used to assess similarities/dissimilarities among the samples, and the latter represents the contribution of each variable for the model decomposition, thus being used to find important spectral markers. This technique looks for inherent similarities/differences and provides a scores matrix representing the overall “identity” of each sample; a loadings matrix representing the spectral profile in each PC; and a residual matrix containing the unexplained data. Scores information can be used for exploratory analysis providing possible classification between data classes.

PCA was the method of choice for analysing swab samples spiked. It is simple, fast and combines exploratory analysis, data reduction and feature extraction into one single method. PCA scores were used to explore overall dataset variance and any clustering related to the limit of detection, while the loadings on the first two PCs were used to derive specific biomarkers indicative of the infection category.

Linear discriminant analysis (LDA) is a classifier with a linear decision boundary, generated by fitting class conditional densities to the data and using Bayes’ rule. The model fits a Gaussian density to each class, assuming that all classes share the same covariance matrix. The fitted model can also be used to reduce the dimensionality of the input by projecting it to the most discriminative directions, using the transform method.

PCA followed by LDA [13] is a combined technique leading to dimensionality reduction followed by classification. Since PCA is an unsupervised dimensionality reduction method, it is difficult to further analyse the differences among groups only from the results of PCA. Therefore, the best classification performance can be demonstrated by applying supervised dimensionality reduction with LDA on the basis of PCA. The combination of PCA and LDA not only reduces the original data dimensionality, maximizes the spectral differences between categories, and improves the accuracy of identifying the differences between groups, but also improves the deficiency of LDA overfitting. The pre-processed spectral data were subsequently subjected to multivariate analysis by PCA-LDA. Herein, PCA was first performed on the spectral data, and 10 principal components (PCs) were extracted from each spectral data (containing >95% of the variable information within the original dataset, which can replace the original variables for LDA), and the extracted PC data were subjected to LDA.

### 2.5. Application of a Machine-Learning Algorithm: K-Nearest Neighbours

The application of k-nearest neighbours (k-NN) was performed using software from scikit-learn. The k-NN is a kernel-based classifier, which requires user-supplied kernel parameters to operate, this being the number of neighbours for k-NN. These parameters should be judiciously selected to ensure effective use of the classifiers in the appropriate kernel complexity avoiding under- and over-fitted operation conditions. Here, it is carried out by minimising the misclassification rate (MR) of the classifier for both the training and testing datasets. This is based on the rationale that, while the MR of the training dataset is expected to continuously fall as the kernel complexity increases, the MR of the testing dataset is expected to reach a minima before it rises as the kernel complexity increases, indicating that it has failed to predict the class of new unseen observations. A high MR for the testing dataset with a low kernel complexity indicates underfitting, whereas a high MR for the testing dataset with a high kernel complexity indicates overfitting. It implies that the suitable kernel complexity occurs at the kernel parameters that result in the minimum MR for the testing dataset [14]. Specific to this work, the range of complexities tested for k-NN was 1 to 400 neighbours. The k-NN models output in the validation set (blind spectra) are used to calculate quality metrics or figures of merit in order to evaluate the model classification performance. Metrics such as accuracy (total number of samples correctly classified considering true and false negatives), sensitivity (proportion of positive observations correctly classified) and specificity (proportion of negative observations correctly classified) are calculated [15].

### 2.6. Statistical Analyses

Multivariate analysis of variance (MANOVA) was performed on PCs using the MANOVA software in python (https://www.statsmodels.org/dev/generated/statsmodels.multivariate.manova.MANOVA.html; accessed on 1 September 2022).

## 3. Results and Discussion

Lung cancer remains the third most common cancer in the UK (CRUK), with rates in Blackpool (North West England) of approximately 160 new cases per year [16]. Whilst rates across England have fallen, those in this region have remained consistently above average [16]. The commonest means by which individuals are diagnosed is through attendance at A&E [17]. As a consequence, Blackpool has recently been selected by NHS England as one of the pilot sites to develop screening for lung cancer. Nesting our study in this prospective screening programme is an ideal means of trialling the clinical translation of this methodology. To our knowledge, this is the first trial of ATR-FTIR spectroscopy with chemometrics in an NHS-ratified and -funded screening programme.

The study contained herein set out to develop a new rapid and non-invasive method of lung cancer detection that could, it is hoped, be used as part of a screening test designed to pick up disease earlier, possibly before an individual has symptoms. This could allow people to be treated earlier, with the aim of improving survival [18]. In line with a developed protocol, patients that were selected for lung cancer screening attended a lung health check clinic. Those that then require a CT scan, based upon the health check results were asked to also take part in this study. Once consented in line with ethics, they provided a sample of saliva in a sterile pot. The saliva was then transported to the laboratory and stored at −20 °C until analysis whereupon a sterile Copan blue rayon swab was dipped into the thawed sample prior to analysis using a hand-held mid-IR spectrometer. The results from this test could be compared to the CT scan performed to see if this new tool can detect cancer-positive patients. As patients without cancer outnumber those with cancer, the use of all patients being scanned is important to provide a large number of non-cancer samples. Another interesting novel aspect of this study is the fact that a genuine clinical setting is employed with samples analysed based on the participants presenting in the programme. Many studies trial the efficacy of test methods using sample collections (e.g., biobanks) and design studies that are equally weighted between control benign and variant (i.e., cancer). In a real-world clinical setting, control benign samples would most typically vastly outweigh the number of variants. This investigation aims to simulate this real-world setting.

Figure 1 shows all the raw IR spectra derived from saliva samples obtained from participants designated either as cancer-free benign or cancer-positive (this included a mixture of lung cancers and metastases to the lung from previous primary cancers). Except for one outlier in a total of 211 study participant saliva samples (*n* = 170 benign control, 41 cancer-positive), remarkable consistency in the spectral signature is noted. For comparison, IR spectra derived from wet (sterile milli-Q water) and dry swabs are shown (five independent swabs, three spectra each). An underlying spectral signature from the swab (wet or dry) is noted. Whilst one approach might be to modify sample preparation to minimise substrate contributions [19], the approach contained herein accepts this on the basis that it is consistent enough so that the overlying bio-fingerprint spectra of the saliva samples provide the variables sufficient for chemometric diagnostics.

**Figure 2:**
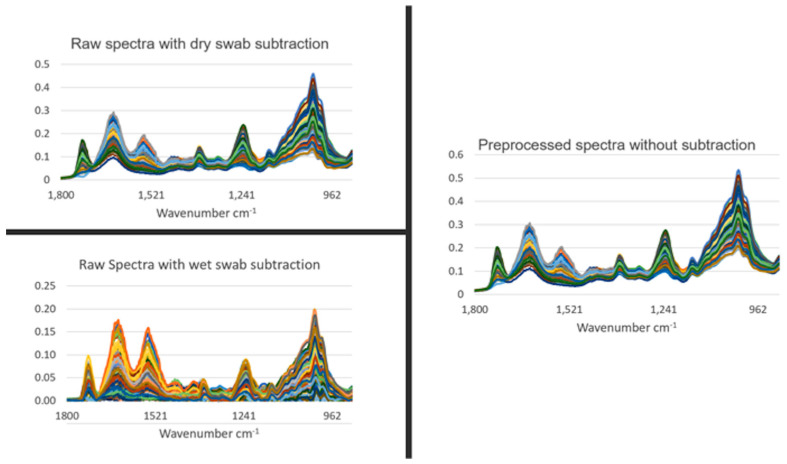
Spectral signatures of saliva samples following pre-processing. From benign control and cancer saliva samples, three independent spectral measurements were taken. Spectral pre-processing consisted of Savitzky–Golay (SG) smoothing (window of 7 points, 1st-order polynomial fitting) and 2nd derivative followed by vector normalization. This was undertaken with or without subtraction of dry or wet (in milli-Q water) control swabs. *y*-axes are Absorbance (a.u.).

Figure 2 shows all the spectra from all the study participants used in this study following spectral pre-processing (Savitzky–Golay (SG) smoothing (window of seven points, first-order polynomial fitting) and second derivative followed by vector normalization) [20]. Despite marked variation, good consistency in spectral appearance is noted. Of significant note is that, following the subtraction of either the wet swab spectra or the dry swab spectra, there remains a marked spectral bio-fingerprint. This strongly suggests that, despite the presence of an underlying swab spectral signature following the mixing in the saliva sample, it remains possible to extract a spectral bio-fingerprint from the study participant. It is this overlying spectral bio-fingerprint that potentially contains the participant features that could indicate the presence or absence of disease.

**Figure 3:**
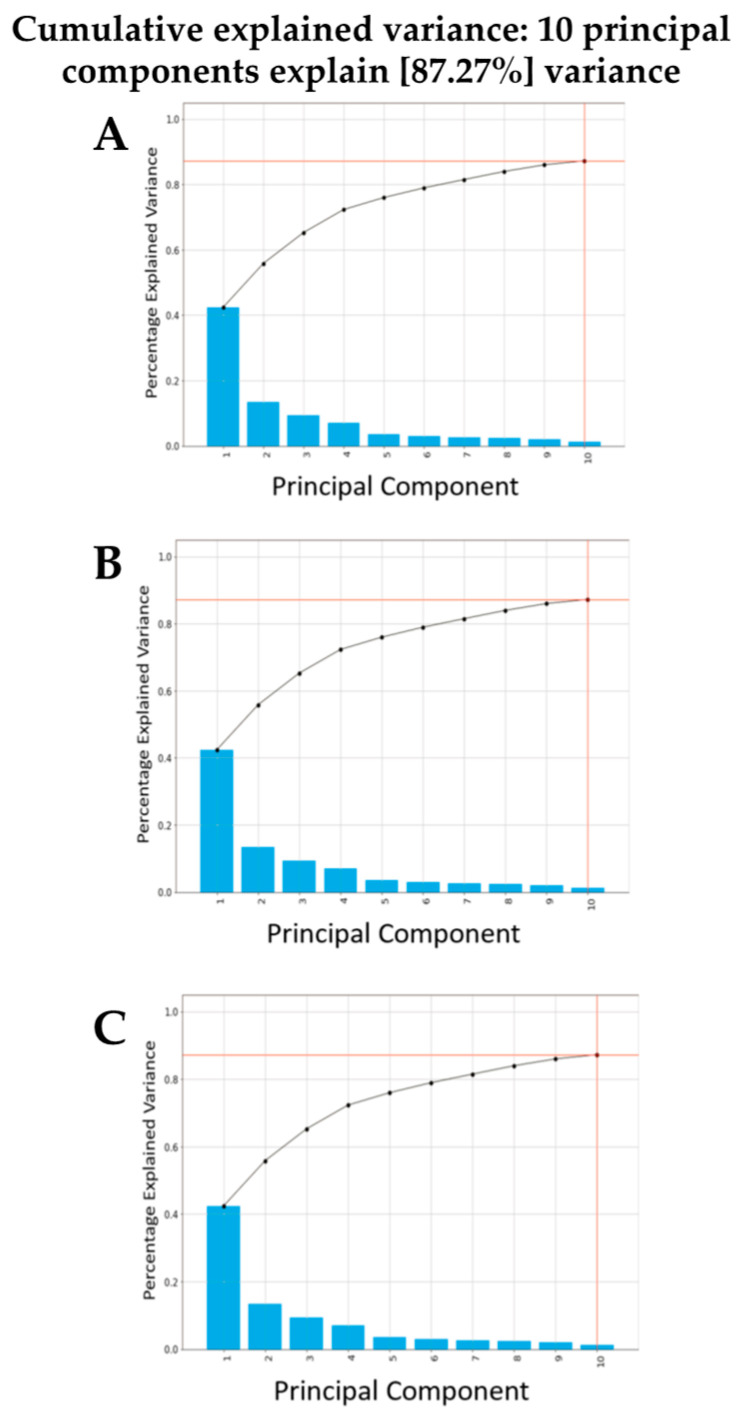
The contribution of the first 10 PCs to variance following PCA. Following pre-processing, a scree plot was constructed for each of the three pairwise comparisons undertaken: (**A**) benign vs. cancer following swab analysis; (**B**) benign vs. cancer following swab analysis with subtraction of dry swab spectra; and (**C**) benign vs. cancer following swab analysis with subtraction of wet swab spectra.

**Figure 4:**
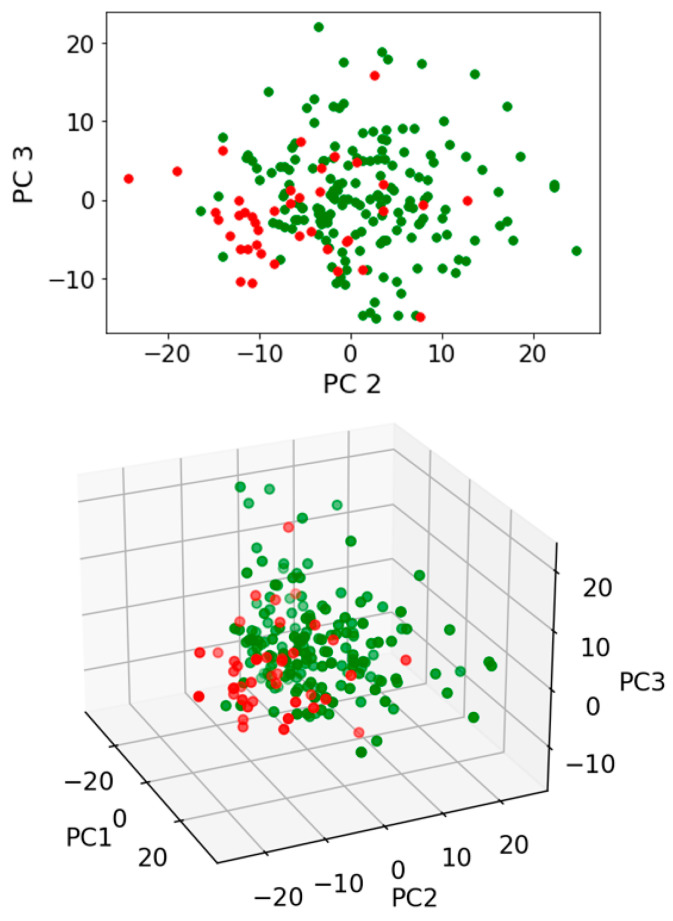
Exploratory analysis of saliva samples on a swab following PCA. Pairwise comparison of benign vs. cancer following swab analysis. PCA scores plots for 2D (plotted on axes for PC2 and PC3) and 3D (plotted on axes for PC1, PC2 and PC3) exploratory analyses are shown. Four independent multivariate analysis of variance (MANOVA) tests were undertaken to test for significance of segregation (see Figure S1). Green spectral points, benign samples; Red spectral points, cancer samples.

**Figure 5:**
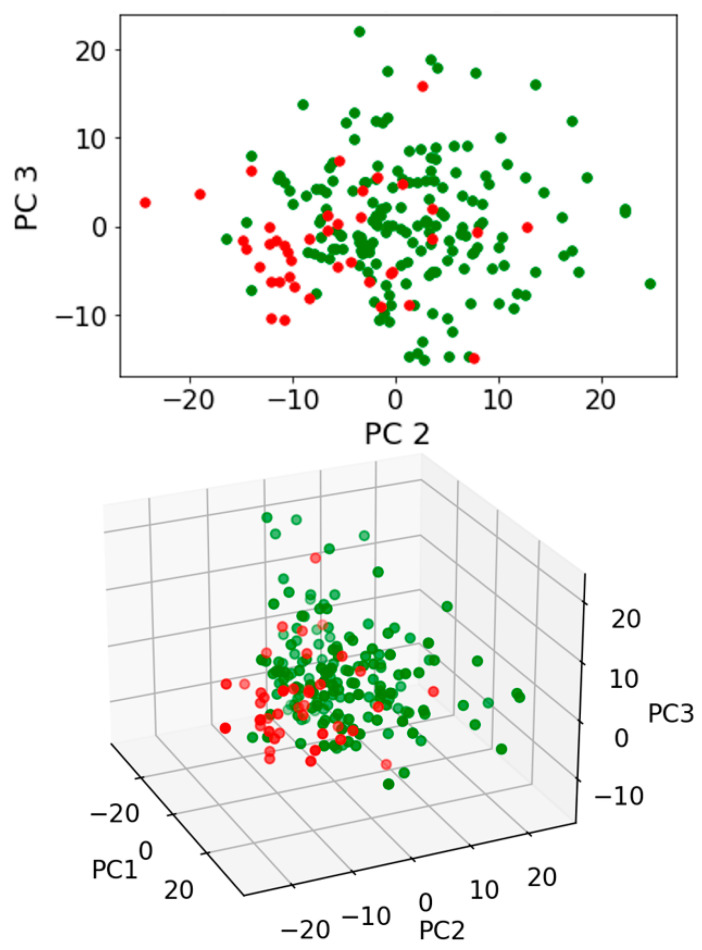
Exploratory analysis of saliva samples minus a dry swab spectral signature following PCA. Pairwise comparisons of benign vs. cancer following swab analysis with subtraction of dry swab spectra. PCA scores plots for 2D (plotted on axes for PC2 and PC3) and 3D (plotted on axes for PC1, PC2 and PC3) exploratory analyses are shown. Four independent multivariate analysis of variance (MANOVA) tests were undertaken to test for significance of segregation (see Figure S2). Green spectral points, benign samples; Red spectral points, cancer samples.

**Figure 6:**
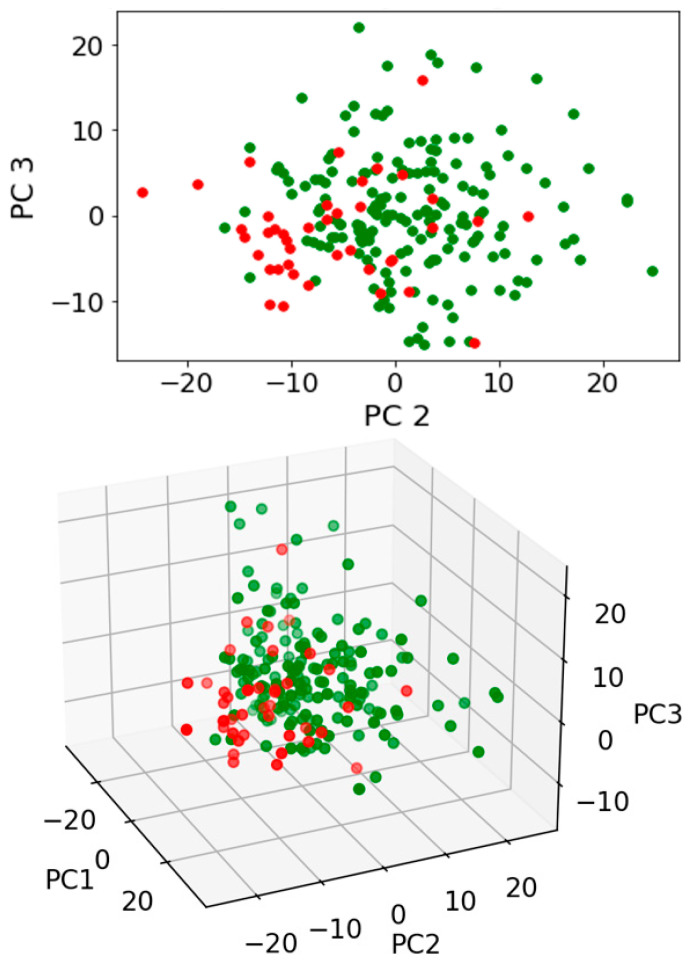
Exploratory analysis of saliva samples minus a wet swab spectral signature following PCA. Pairwise comparisons of benign vs. cancer following swab analysis with subtraction of wet swab spectra. PCA scores plots for 2D (plotted on axes for PC2 and PC3) and 3D (plotted on axes for PC1, PC2 and PC3) exploratory analyses are shown. Four independent multivariate analysis of variance (MANOVA) tests were undertaken to test for significance of segregation (see Figure S3). Green spectral points, benign samples; Red spectral points, cancer samples.

Exploratory analysis using PCA with or without LDA was then undertaken. Three pairwise comparisons were conducted including: (1) benign vs. cancer following swab analysis; (2) benign vs. cancer following swab analysis with the subtraction of dry swab spectra; and (3) benign vs. cancer following swab analysis with the subtraction of wet swab spectra. Within this prospective study of people invited to attend the National Lung Cancer screening pilot in the Blackpool area of North West England, we used only those patients who had had a CT scan, in order to ensure a comparison. Those with positive CT scans were followed up via clinical records. Most had confirmed histology; others were treated based on scan appearances following a multidisciplinary team (MDT) discussion. Either was taken as a diagnosis of cancer, as this was the information provided to the patient. Figure 3 shows the contributions to variance of each of the first 10 PCs in each pairwise comparison. Remarkable similarity is noted for each pairwise comparison, irrespective of benign vs. cancer following swab analysis (Figure 3A); benign vs. cancer following swab analysis with the subtraction of dry swab spectra (Figure 3B); or benign vs. cancer following swab analysis with the subtraction of wet swab spectra (Figure 3C). Figure 4, Figure 5 and Figure 6 then show the PCA scores plots for 2D (plotted on axes for PC2 and PC3) and 3D (plotted on axes for PC1, PC2 and PC3) exploratory analyses. These show a consistent and remarkably similar pattern of clustering for the benign control vs. cancer categories, irrespective of whether the swab plus saliva sample is analysed or whether there is a subtraction for the wet or dry swab. The critical aspect of this observation is that the blue rayon swab as a substrate does not appear to influence the profile of the output results. In all cases also, MANOVA points to marked significance in benign control vs. cancer samples (see Supplementary Information Figures S1–S3). The separation between the categories is also examined by employing PCA-LDA (using the first 10 PCs) in a 1D scores plot (Figures S1–S3). Herein, the crossover samples (*n* = 14 aligning with benign controls) are more readily identifiable. An examination of the study participant demographics did not highlight any consistency in the profiles of these crossover samples; some were early-stage lung cancers, others were metastases and one had two cancers (a primary lung cancer and a metastasis). However, again the pattern of separation is remarkably similar for the benign control vs. cancer categories, irrespective of whether swab plus saliva sample is analysed or whether there is a subtraction for the wet or dry swab.

**Figure 7:**
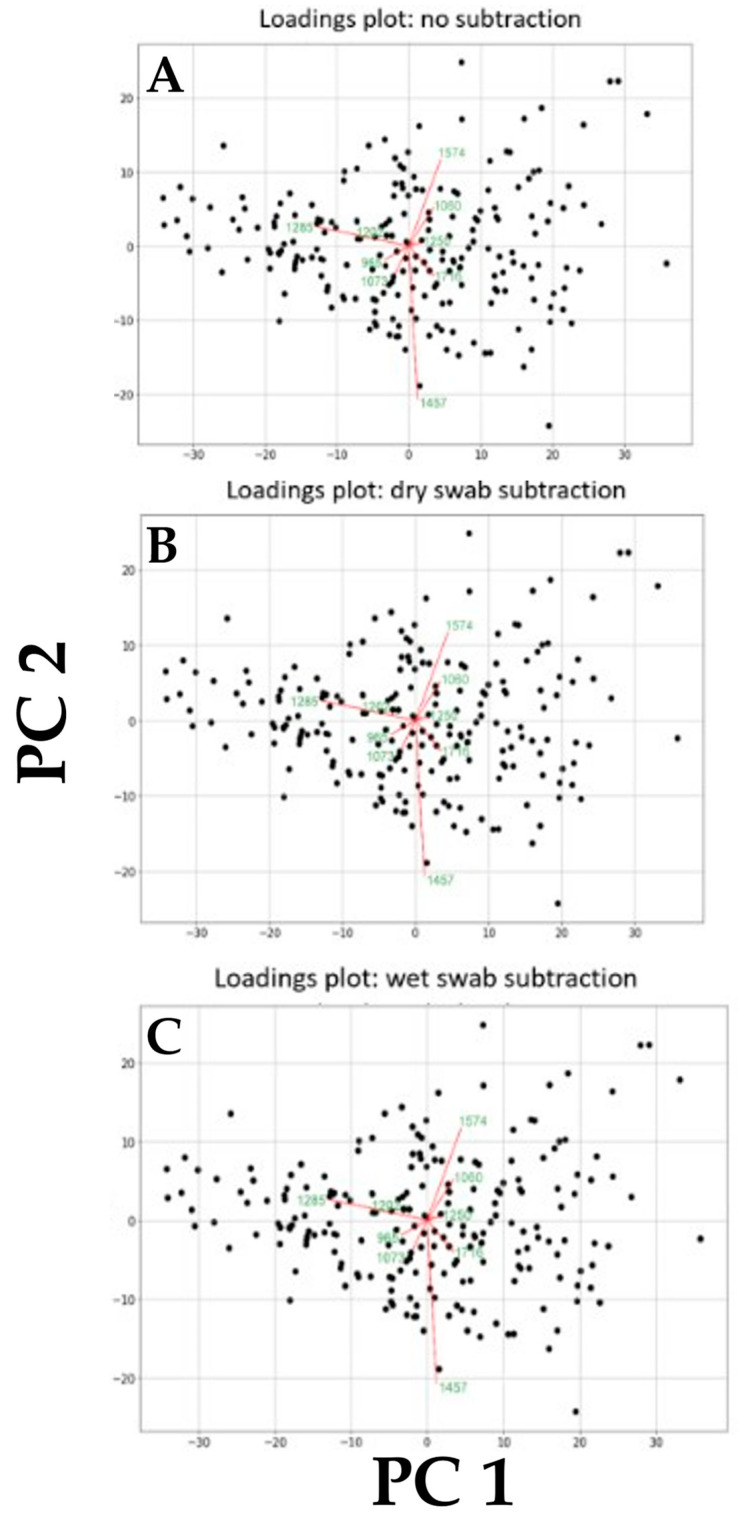
Loadings plots exhibiting the wavenumber contributing the most to variance along each of the first 10 PCs. These were obtained following pairwise comparisons including: (**A**) benign vs. cancer following swab analysis; (**B**) benign vs. cancer following swab analysis with subtraction of dry swab spectra; and (**C**) benign vs. cancer following swab analysis with subtraction of wet swab spectra.

Figure 7 shows the most important wavenumbers responsible for separation along the first 10 PCs for the three pairwise comparisons that were undertaken including: (A) benign control vs. cancer following swab analysis; (B) benign control vs. cancer following swab analysis with the subtraction of dry swab spectra; and (C) benign control vs. cancer following swab analysis with the subtraction of wet swab spectra. The loadings plots demonstrate how strongly a spectral wavenumber influences a PC (see Figure S4). For each comparison, these are remarkably similar. This, again, lends further weight to our hypothesis that a sterile blue rayon swab is an ideal substrate to allow for a dip test in a biofluid such as saliva, which can then be readily and consistently applied to the sensor on the IR spectrometer. This is a critical observation because, if there was a lack of consistency in the background swab spectral signature, this could conceivably introduce a level of variance so great as to increase the difficulty of extracting the features responsible for discriminating between benign control and cancer in this screening approach. The extraction of important biological information, despite the presence of contaminating spectral peaks (e.g., paraffin wax from histological blocks), has been achieved in previous studies [21,22].

**Figure 8:**
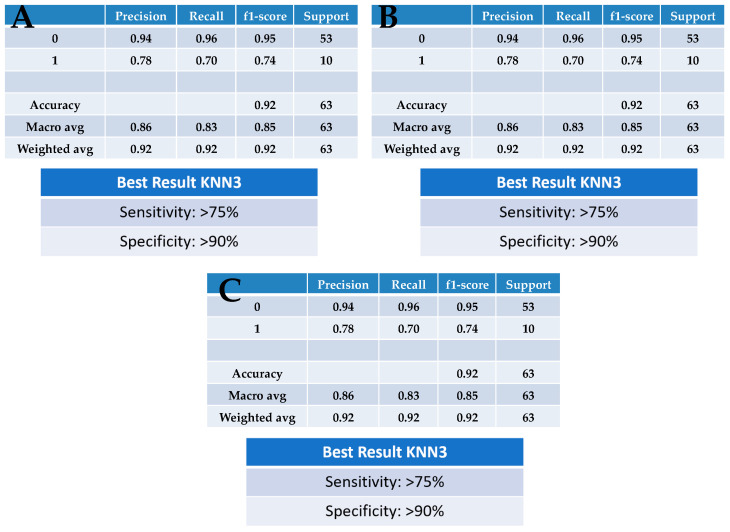
Application of k-nearest neighbours (k-NN) as a machine-learning algorithm. Employing a three nearest neighbours construction, this was undertaken for each of the three pairwise comparisons undertaken: (**A**) benign vs. cancer following swab analysis; (**B**) benign vs. cancer following swab analysis with subtraction of dry swab spectra; and (**C**) benign vs. cancer following swab analysis with subtraction of wet swab spectra.

Figure 8 shows the results from a k-NN (using three nearest neighbours) machine-learning algorithm; the use of such machine-learning algorithms in lung cancer screening is becoming popular [23,24,25]. A three nearest neighbours construction was employed for each pairwise comparison (see Figure S5). The specificity (90%) and sensitivity (75%) obtained using k-NN are consistent for each pairwise comparison undertaken using PCA or PCA-LDA: (A) benign vs. cancer following swab analysis; (B) benign vs. cancer following swab analysis with the subtraction of dry swab spectra; and (C) benign vs. cancer following swab analysis with the subtraction of wet swab spectra. Although this is a pilot sampling of a larger project, this points to a test that would already be acceptable within a clinical setting. This would fit well with the requirements of a lung-cancer-screening test where one also wants to maximise benefits and minimise harm [26]. In an aging population, it is becoming increasingly difficult to carry out extensive investigations using, for instance, imaging techniques or molecular markers. A rapid and reagent-free test, that is inexpensive, readily repeatable and equally applicable to extensive point-of-care testing as a triage-screening tool, is hugely attractive. It is critical to consider the financial pressures and lack of medical resources in the implementation of a screening intervention programme [27]; the ability to roll out a test to the general population and to have the structure for a recall system emphasises the need for a simplistic testing approach.

This study set out to establish whether a sterile blue rayon swab could be used as a substrate in a dip test for saliva samples obtained from participants in a lung cancer-screening programme. Although the swabs themselves (wet or dry) exhibit a background spectral signature in the bio-fingerprint mid-IR region, this was insufficient to negate objective benign control vs. cancer discrimination. Remarkably similar discrimination is noted, with or without subtraction of wet or dry swab spectral signatures, following exploratory analyses. Lung cancer has traditionally affected smokers of an older demographic. Its prevalence is higher in areas of deprivation due to its association with smoking and, as symptoms may be vague or non-existent, in its early stages it can often present late [28]. With the advent of molecular pathology, genomic profiling of tumours has led to personalised treatments, with huge increases in survival, even for metastatic patients [29,30]. The optimal lung pathway, therefore, provides time points at which a person should be diagnosed, profiled and referred for treatment in order to optimise outcomes [31].

This study is part of a larger prospective screening programme where a power calculation has been undertaken. The small sample size is a limitation herein, but we expect this to be addressed as the screening programme progresses. Saliva as a liquid biopsy confers many advantages in terms of its ease of acquisition and the non-invasive nature in which it can be obtained. There is no need for a Research Nurse to take blood samples from a patient (which, in older people, might be difficult), nor is there a need for a processing laboratory if serum or plasma is needed. The major limitation is that one is relying on surrogate biomarkers of disease to be present in the saliva. With growing evidence that the oral cavity can be an indicator of overall health, evidence in this study points to the fact that saliva might also indicate the presence or absence of lung cancer.

Even in this pilot study, exploratory analyses points to a test that already exhibits adequate sensitivity and specificity for a point-of-care clinical setting. The standardisation of this approach in a multi-centre trial would also be required [32]. Saliva appears to be an increasingly promising liquid biopsy for cancer screening [33]. Equally, it appears that a sterile swab (which many healthcare professionals will be familiar with) can be used as a substrate to conveniently and safely apply this readily attainable liquid biopsy to the ATR crystal in order to obtain a fingerprint spectrum. Harnessed to chemometric and machine-learning algorithms, this approach has enormous potential as a rapid screening and triage tool in point-of-care clinical settings. We now propose to roll this screening approach out to the entire population, screening under this programme to ascertain its performance in comparison with methods such as low-dose CT scans.

## Data Availability

Data contributing to this manuscript will be made available upon reasonable request to the corresponding authors.

## Supplementary Materials

The following supporting information can be downloaded at: https://www.mdpi.com/article/10.3390/jpm13071039/s1, Figure S1: Exploratory analysis of saliva samples on a swab following PCA. Following pairwise comparisons of benign versus cancer, four independent multivariate analysis of variance (MANOVA) tests were undertaken to test for significance of segregation. Separation between the categories is also examined employing PCA-LDA (using the first 10 PCs) in a 1D scores plot.; Figure S2: Exploratory analysis of saliva samples, minus a dry swab spectral signature, following PCA. Following pairwise comparisons of benign versus cancer, four independent multivariate analysis of variance (MANOVA) tests were undertaken to test for significance of segregation. Separation between the categories is also examined employing PCA-LDA (using the first 10 PCs) in a 1D scores plot.; Figure S3: Exploratory analysis of saliva samples, minus a wet swab spectral signature, following PCA. Following pairwise comparisons of benign versus cancer, four independent multivariate analysis of variance (MANOVA) tests were undertaken to test for significance of segregation. Separation between the categories is also examined employing PCA-LDA (using the first 10 PCs) in a 1D scores plot.; Figure S4: The wavenumbers contributing the most to variance along each of the first 10 PCs (87.2% total variance). Obtained following pairwise comparisons: (A) benign versus cancer following swab analysis; (B) benign versus cancer following swab analysis with subtraction of dry swab spectra; and, (C) benign versus cancer following swab analysis with subtraction of wet swab spectra.; Figure S5: Application of k-nearest neighbours (k-NN). A three nearest neighbours construction was undertaken for each of the three pairwise comparisons undertaken: (A) benign versus cancer following swab analysis; (B) benign versus cancer following swab analysis with subtraction of dry swab spectra; and (C) benign versus cancer following swab analysis with subtraction of wet swab spectra.
