# Supplementary material for: ATR-FTIR Spectroscopy with Chemometrics for Analysis of Saliva Samples Obtained in a Lung-Cancer-Screening Programme: Application of Swabs as a Paradigm for High Throughput in a Clinical Setting
Source: J Pers Med. 2023 Jun 25;13(7):1039. doi: 10.3390/jpm13071039 (PMC10381591; doi:10.3390/jpm13071039)
Supplement: Supplementary file 1 [file jpm-13-01039-s001.zip › jpm-2421923-supplementary.pdf]

Electronic Supplementary Information

ATR-FTIR Spectroscopy with Chemometrics for Analysis of Saliva Samples Obtained in a Lung-Cancer-Screening Programme: Application of Swabs as a Paradigm for High Throughput in a Clinical Setting

Francis L Martin [1,2,*], Andrew W Dickinson [2], Tarek Saba [2], Thomas Bongers [2], Maneesh N Singh [1,3], Danielle Bury [1,*]

1 Biocel UK Ltd., Hull HU10 6TS, UK; mnsingh@biocel.uk
2 Department of Cellular Pathology, Blackpool Teaching Hospitals NHS Foundation Trust, Whinney Heys Road, Blackpool FY3 8NR, UK; aw.dickinson@hotmail.com (A.W.D.); dr.saba@nhs.net (T.S.); thomas.bongers@nhs.net (T.B.)
3 Chesterfield Royal Hospital, Chesterfield Road, Calow, Chesterfield S44 5BL, UK
* Correspondence: flm13@biocel.uk (F.L.M.); danielle.bury@nhs.net (D.B.)

Multivariate linear model

PC2 vs PC3 MANOVA — **No Subtraction**

Intercept	Value	Num DF	Den DF	F Value	Pr > F
Wilks' lambda	0.9622	2.0000	207.0000	4.0651	0.0185
Pillai's trace	0.0378	2.0000	207.0000	4.0651	0.0185
Hotelling-Lawley trace	0.0393	2.0000	207.0000	4.0651	0.0185
Roy's greatest root	0.0393	2.0000	207.0000	4.0651	0.0185

Diagnosis	Value	Num DF	Den DF	F Value	Pr > F
Wilks' lambda	0.8325	2.0000	207.0000	20.8212	0.0000
Pillai's trace	0.1675	2.0000	207.0000	20.8212	0.0000
Hotelling-Lawley trace	0.2012	2.0000	207.0000	20.8212	0.0000
Roy's greatest root	0.2012	2.0000	207.0000	20.8212	0.0000

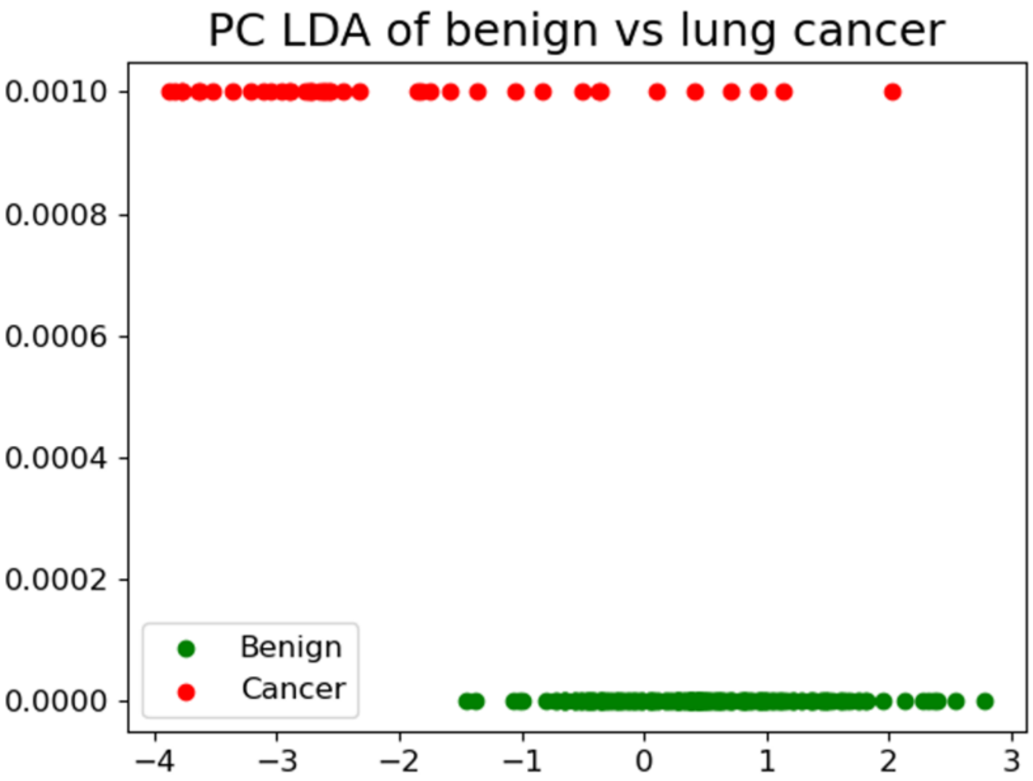

Figure S1 Exploratory analysis of saliva samples on a swab following PCA. Following pairwise comparisons of benign *versus* cancer, four independent multivariate analysis of variance (MANOVA) tests were undertaken to test for significance of segregation. Separation between the categories is also examined employing PCA-LDA (using the first 10 PCs) in a 1-D scores plot.

Multivariate linear model

PC2 vs PC3 MANOVA — **Dry swab subtracted**

Intercept	Value	Num DF	Den DF	F Value	Pr > F
Wilks' lambda	0.9622	2.0000	207.0000	4.0651	0.0185
Pillai's trace	0.0378	2.0000	207.0000	4.0651	0.0185
Hotelling-Lawley trace	0.0393	2.0000	207.0000	4.0651	0.0185
Roy's greatest root	0.0393	2.0000	207.0000	4.0651	0.0185

Diagnosis	Value	Num DF	Den DF	F Value	Pr > F
Wilks' lambda	0.8325	2.0000	207.0000	20.8212	0.0000
Pillai's trace	0.1675	2.0000	207.0000	20.8212	0.0000
Hotelling-Lawley trace	0.2012	2.0000	207.0000	20.8212	0.0000
Roy's greatest root	0.2012	2.0000	207.0000	20.8212	0.0000

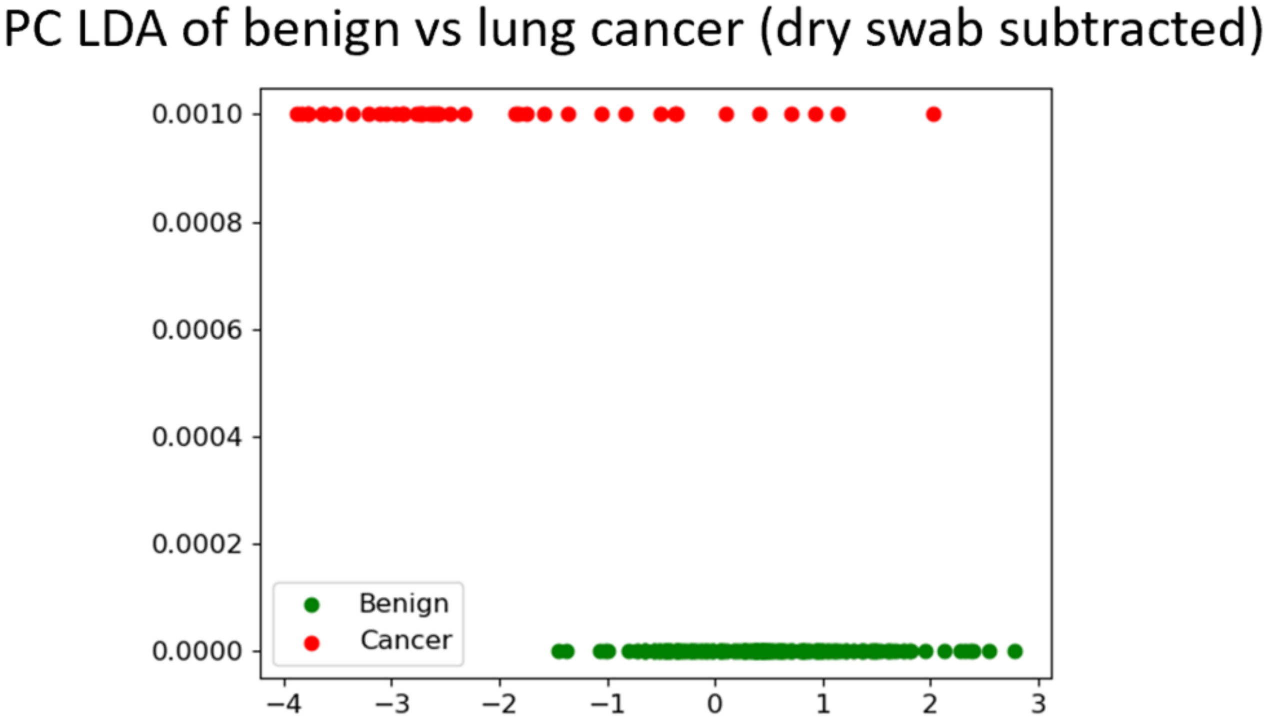

Figure S2 Exploratory analysis of saliva samples minus a dry swab spectral signature following PCA. Following pairwise comparisons of benign *versus* cancer, four independent multivariate analysis of variance (MANOVA) tests were undertaken to test for significance of segregation. Separation between the categories is also examined employing PCA-LDA (using the first 10 PCs) in a 1-D scores plot.

Multivariate linear model

PC2 vs PC3 MANOVA **Wet swab subtracted**

Intercept	Value	Num DF	Den DF	F Value	Pr > F
Wilks' lambda	0.9622	2.0000	207.0000	4.0651	0.0185
Pillai's trace	0.0378	2.0000	207.0000	4.0651	0.0185
Hotelling-Lawley trace	0.0393	2.0000	207.0000	4.0651	0.0185
Roy's greatest root	0.0393	2.0000	207.0000	4.0651	0.0185

Diagnosis	Value	Num DF	Den DF	F Value	Pr > F
Wilks' lambda	0.8325	2.0000	207.0000	20.8212	0.0000
Pillai's trace	0.1675	2.0000	207.0000	20.8212	0.0000
Hotelling-Lawley trace	0.2012	2.0000	207.0000	20.8212	0.0000
Roy's greatest root	0.2012	2.0000	207.0000	20.8212	0.0000

PC LDA of benign vs lung cancer (wet swab subtracted)

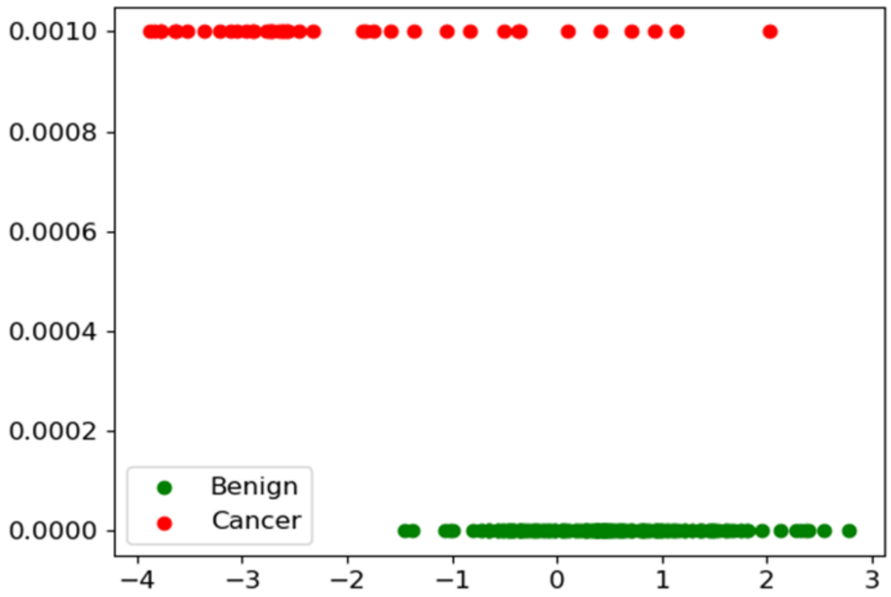

Figure S3 Exploratory analysis of saliva samples minus a wet swab spectral signature following PCA. Following pairwise comparisons of benign *versus* cancer, four independent multivariate analysis of variance (MANOVA) tests were undertaken to test for significance of segregation. Separation between the categories is also examined employing PCA-LDA (using the first 10 PCs) in a 1-D scores plot.

A

B

C

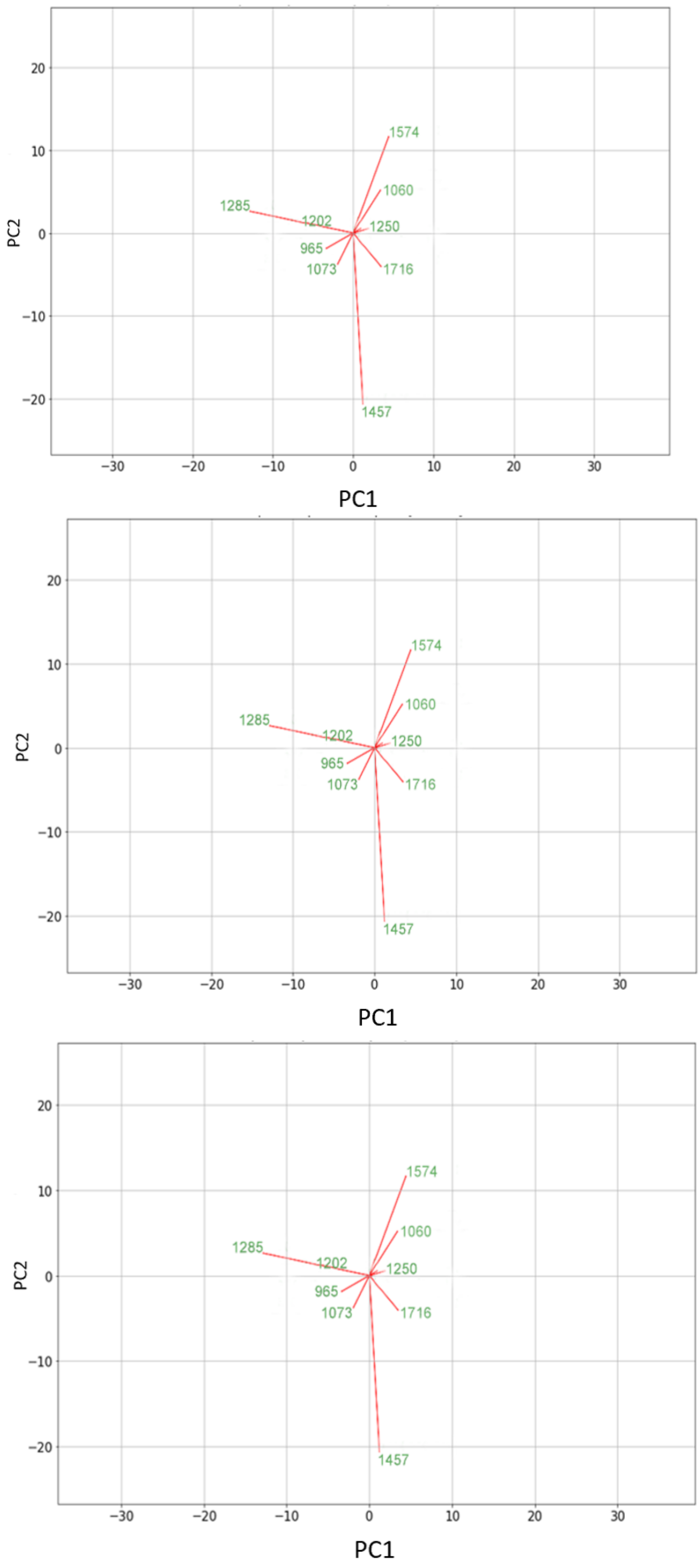

Figure S4 The wavenumbers contributing the most to variance along each of the first 10 PCs (87.2% total variance). Obtained following pairwise comparisons: A) benign *versus* cancer following swab analysis; B) benign *versus* cancer following swab analysis with subtraction of dry swab spectra; and, C) benign *versus* cancer following swab analysis with subtraction of wet swab spectra.

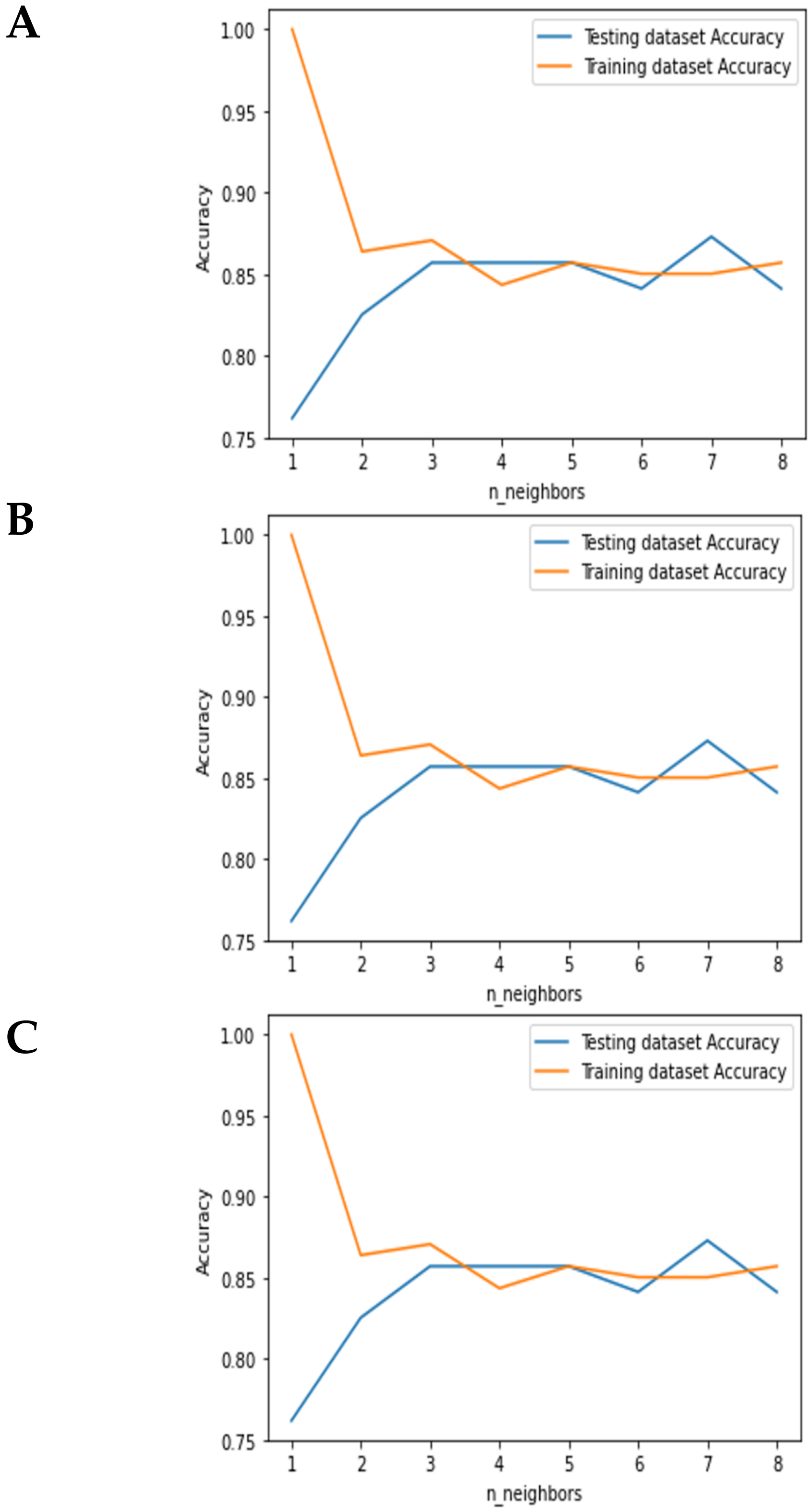

Figure S5 Application of k-nearest neighbours (k-NN). A three nearest neighbours construction was undertaken for each of the three pairwise comparisons undertaken: A) benign *versus* cancer following swab analysis; B) benign *versus* cancer following swab analysis with subtraction of dry swab spectra; and, C) benign *versus* cancer following swab analysis with subtraction of wet swab spectra.
